# Ferrocene Orientation Determined Intramolecular Interactions Using Energy Decomposition Analysis

**DOI:** 10.3390/ma8115419

**Published:** 2015-11-16

**Authors:** Feng Wang, Shawkat Islam, Vladislav Vasilyev

**Affiliations:** 1 Molecular Model Discovery Laboratory, Department of Chemistry and Biotechnology, Faculty of Science, Engineering and Technology, Swinburne University of Technology, Hawthorn, Melbourne 3122, Australia; sislam@swin.edu.au; 2 National Computational Infrastructure, Australian National University, Canberra 0200, Australia; vvv900@gmail.com

**Keywords:** ferrocene, eclipsed and staggered conformers, energy decomposition analysis, natural bond orbital scheme, intramolecular interaction, quantum mechanical models

## Abstract

Two very different quantum mechanically based energy decomposition analyses (EDA) schemes are employed to study the dominant energy differences between the eclipsed and staggered ferrocene conformers. One is the extended transition state (ETS) based on the Amsterdam Density Functional (ADF) package and the other is natural EDA (NEDA) based in the General Atomic and Molecular Electronic Structure System (GAMESS) package. It reveals that in addition to the model (theory and basis set), the fragmentation channels more significantly affect the interaction energy terms (Δ*E*) between the conformers. It is discovered that such an interaction energy can be absorbed into the pre-partitioned fragment channels so that to affect the interaction energies in a particular conformer of Fc. To avoid this, the present study employs a complete fragment channel—the fragments of ferrocene are individual neutral atoms. It therefore discovers that the major difference between the ferrocene conformers is due to the quantum mechanical Pauli repulsive energy and orbital attractive energy, leading to the eclipsed ferrocene the energy preferred structure. The NEDA scheme further indicates that the sum of attractive (negative) polarization (POL) and charge transfer (CL) energies prefers the eclipsed ferrocene. The repulsive (positive) deformation (DEF) energy, which is dominated by the cyclopentadienyle (Cp) rings, prefers the staggered ferrocene. Again, the cancellation results in a small energy residue in favour of the eclipsed ferrocene, in agreement with the ETS scheme. Further Natural Bond Orbital (NBO) analysis indicates that all NBO energies, total Lewis (no Fe) and lone pair (LP) deletion all prefer the eclipsed Fc conformer. The most significant energy preferring the eclipsed ferrocene without cancellation is the interactions between the donor lone pairs (LP) of the Fe atom and the acceptor antibond (BD*) NBOs of all C–C and C–H bonds in the ligand, LP(Fe)-BD*(C–C & C–H), which strongly stabilizes the eclipsed (D_5h_) conformation by −457.6 kcal·mol^−1^.

## 1. Introduction

Since its discovery, ferrocene [1], *i.e.*, di-cyclopentadienyle iron (η^5^, FeCp_2_ or Fc), has been extensively studied experimentally and theoretically with exhausting information. The ground-electronic state conformation of Fc due to the orientation of two parallel cyclopentadienyl (Cp) rings of Fc, which gives the eclipsed (E) or staggered (S) conformers, is surprisingly difficult to resolve unambiguously in both theory [2,3] and experiment [4,5,6,7,8] A number of challenges are manifest in the studies of Fc since its discovery [1]. Firstly, the extremely small energy difference between the conformers, *ca.* 0.9 ± 0.3 kcal·mol^−1^, using electron diffraction in gas phase [4], and the calculated energy of 0.58 kcal·mol^−1^ using the B3LYP/m6-31G(d) model [2,3] is likely within the error bars of many methods including quantum mechanical methods. Secondly, the low energy barrier for the rotation of a cyclopentadienyl ring relative to the rest of the molecule [9,10] makes the problem more complex; and finally, lack of permanent dipole moment due to very high point group symmetry for both conformers, D_5h_ and D_5d_ further complexes the issue [3].

Detailed structural understanding of the Fc conformers is very important as Fc derivatives may inherit particular properties that only exist in a particular conformer [10]. For example, additional ligand coordinating to the metal and the Cp rings while maintaining certain symmetry is preferred for the geometry of the D_5h_ conformer [11,12,13]. Design of synthesis pathways and understanding of the mechanics and reaction dynamics of the Fc derivatives such as catalysts require detailed information of the structure, symmetry and properties of the Fc conformers. The stability of the eclipsed and staggered conformers of Fc has been a challenge issue, and both structures were discussed in textbooks [14,15,16] presenting ferrocene. A number of recent articles such as Duhović and Diaconsecu [11], Coriani *et al.* [17], Roy *et al.* [18], Gryaznova *et al.* [13] Bean *et al.* [19] Frenking and co-workers [20,21,22,23,24] and Cortés-Guzmán and Bader [25] have well documented the history and current status of Fc studies. The debate on the most stable conformer of Fc, whether it is the eclipsed (E) or the staggered (S), however, remains. Recently, the signature of the eclipsed Fc has been determined using infrared (IR) spectral calculations [2].

Structures and properties of the staggered (S) and eclipsed (E) conformers of Fc are markedly similar. As indicated by Coriani *et al.* [17], Fc is a “notoriously difficult example”, as it contains transition metal Fe, which leads to much larger errors because of more complex bonding situations and its d-electrons. Although many recent quantum mechanical studies using high level post-HF methods, such as Møller-Plesset perturbation theory (MP2), coupled-cluster singles/doubles (CCSD) and coupled-cluster triples CCSD(T) in combination with a number of large basis sets such as TZV2P+f [17] and using various Density Functional Theory (DFT) models including BHLP, B3LYP, BLYP, BP86, local spin-density approximation (LSDA) [26] and BPW91 [13] models, the puzzle of E-Fc or S-Fc remains. Nevertheless, all consistently suggest that the eclipsed Fc exists in equilibrium as the minimum structure of Fc [2,17,18,20,27]. Studies using the B3LYP/m6-31G(d) model indicated that in addition to the quantum mechanical method, the basis set needs to including more d-functions of Fe [2,3].

The differences between most molecular properties of the Fc conformers, such as energetics, Fe-Cp bond length, rotational constants and total electronic energies *etc.* are almost identical [2], in addition to the small Jahn-Teller effect [28]. Hence, many studies of Fc conformers are largely contradictory and depending on the models and measurement means [29,30,31]. As a result, either E-Fc or S-Fc has been arbitrarily employed to present the structure of Fc. Our recent DFT based infrared (IR) study of Fc revealed that the IR spectral splitting within the region of 400–500 cm^−1^ serves as the signature region for E-Fc conformer in gas phase [2]. It was measured in a number of experimental IR spectra including the early IR measurements of Fc of Lippincott and Nelson [7,8,32], although the signature of ferrocene eclipsed conformer lacked recognition in the past.

The high thermal stability of ferrocene (upto 400 °C) indicates that the chemical bonding of the complex is special as transition metal-carbon bonds are usually unstable [28]. In order to understand the unique interactions within the E-Fc and the S-Fc, more detailed electronic structural studies of ferrocene conformers are needed. In this regard, theoretical study has its own advantages. In a recent study [3], we introduced an excess orbital energy spectrum (EOES) of ferrocene conformers (E-Fc and S-Fc), in order to identify the differences between the conformers and their unique chemical bonding on the molecular orbital base. It is discovered that the orbitals of Fc, core and valence, which are dominated by the transition metal Fe, show large differences between the conformers of Fc [3].

Dividing the total molecular energy of Fc into orbital energies using molecular orbital theory is an important method of studying a molecule. There are other ways to study energy contributions of a molecule. It is analogous to a number of manners of cake-cutting. The present study intends to decompose the energy contributions of the conformers using the energy decomposition analysis (EDA) [33,34,35]. Significant work with various methods has been done in this direction for ferrocene. For example, Frenking and coworkers [20,21,22] have done significant work on energy decomposition analysis of ferrocene. Others, such as Gomez-Sandoval *et al.* [36] Swart [37], Zhang *et al.* [38], *etc.* also studied ferrocene from different viewpoint, *i.e.*, using different conformers, or different fragmentation schemes or different models and basis sets (either Gaussian basis sets or Slater basis sets), which provide excellent additional information of Fc. However, it seems that a direct comparison can be difficult, as the calculated energy components depend on multiple factors. Firstly, the energies are fragmentation channel dependent, such as heterolytic and homolytic [21]. The component energies are significantly different from different fragmentation schemes. Secondly, the energies are model dependent, both methods and basis sets. For example, the energies calculated using HF, BP86, *etc.* are very different, as are the basis sets. Thirdly, most of such EDA studies concentrate on the D_5d_ staggered conformer [20,21,22,36,38], very few on the eclipsed Fc [37,38,39]. Finally, most of the EDA studies concentrate on the extended transition state (ETS) [33,35] based on the ADF computational package. In the present study, we focus on the differences of the decomposed energies between the eclipsed and staggered conformers, using the atomic fragment scheme. This individual atomic fragment scheme does not prefer a particular conformer or fragmentation scheme. In addition, two different EDA schemes, the extended transition state (ETS) [33,35] based on the ADF computational chemistry package [40] and the natural energy decomposition analysis (NEDA) [34,41,42,43] based on the GAMESS package [44] are employed in the present study.

## 2. Computational Methods and Details

Any decomposition of the interaction energy into separate terms is artificial, so in principle one cannot go wrong when choosing a particular decomposition scheme [45]. Electron configuration or orbitals of a molecule is one way to understand the energy distributions of the conformers, based on the one-particle approximation or the molecular orbital theory. The recent orbital based excess orbital energy spectra (EOES) [3] indeed reveal apparent orbital energy differences of the ferrocene conformers. Most of those orbitals with excess orbital energies are dominated by the Fe electrons not only in the valence space, but also deep in the core space [3]. There are certainly other ways to decompose the energy of a molecule, which will provide significantly different physical insight to help one to understand the conformers of Fc from a different aspect. As indicated by Rayón and Frenking [21], it can be helpful if the results of the orbital analysis were combined with EDA, which also considers electrostatic bonding.

Energy decomposition analysis (EDA) is a very useful tool to dissect the physical origins of intra- or intermolecular interactions of molecules [41,46,47,48,49,50,51,52,53,54,55,56]. It is a powerful method that bridges the gap between elementary quantum mechanics and a conceptually simple interpretation of the nature of the chemical bond [46]. The most well-known EDA is based on Morokuma [35] and the extended transition state (ETS) of Ziegler and Rauk [33]. It has been extended to base on different physical models to decompose the energies of a molecule [41,57,58,59,60,61]. The present study will concentrate on two EDA schemes: one is the ADF based ETS scheme [33] and the other is the GAMESS based NEDA scheme [34,41,42,43].

In the ETS method [33,35], the total bonding energy of the fragments is expressed as the sum between two energy terms at the equilibrium [36]:

Δ*E* = Δ*E_Prep_* + Δ*E_Int_* (1)

The first contribution, Δ*E_Prep_*, is the preparation energy and corresponds to the energy required to deform the separated fragments from their equilibrium geometry to the one they assume in the supermolecule. The second term is a stabilizing term called interaction energy, Δ*E_Int_*, which includes the instantaneous interaction between the fragments. It can be considered as the difference between the energy of the molecule and the energies of the prepared fragments [46]. The Δ*E_Int_* term itself can be decomposed into three quantities that have a direct physical meaning [23,40,45]:

Δ*E_Int_* = Δ*E_Pauli_* + Δ*E_Elstat_* + Δ*E_Orb_* (2)

The first term, Δ*E_Pauli_*, is computed by enforcing the Kohn-Sham determinant of the molecule, which results from superimposing fragments to obey the Pauli principle by ortho-normalizing and anti-symmetrizing the fragment spin-orbitals of one moiety with the fragment spin-orbitals of the second moiety at the supermolecule equilibrium position. This step ensures that same spin electrons do not occupy the same region of space. Usually this term is responsible for the increase in kinetic energy upon formation of a chemical bond and is always positive. The second term, Δ*E_Elstat_*, is computed as the electrostatic interaction between the unperturbed fragments at the equilibrium position. While Δ*E_Pauli_* is repulsive and ΔE_Elstat_ is usually attractive, superposition of these two terms can be called steric energy [62,63,64,65] where

Δ*E_Steric_* = Δ*E_Pauli_* + Δ*E_Elstat_* (3)

The last term, Δ*E_Orb_*, in Equation (1) is computed by relaxing the ortho-normalized density to the fully optimized electron density of the entire supermolecule. This contribution includes the charge transfer between the occupied orbitals of one fragment and the unoccupied orbitals of the other fragments. It also includes charge transfer within the occupied and unoccupied orbitals of the same fragment, *i.e.*, the intra-fragment polarization. The Δ*E_Orb_* term is always attractive as the total wave function is optimized during its calculation. More specifically, it allows the virtual orbitals on the fragments to be mixed in, and therefore includes highest occupied molecular orbital (HOMO)-lowest unoccupied molecular orbital (LUMO) interactions [45]. In addition, it is possible to decompose the orbital interaction Δ*E_Orb_* term further into contributions from orbitals, which belong to different irreducible representations of the point group of the molecule.

In the natural bond orbital (NBO) concept, it constructs a unique set of atomic hybrids and bond orbitals for a given molecule. It uses the information contained in the (exact or approximate) first-order density matrix [66], thereby constructing its "Lewis structure" in an *a priori* manner. Thus, NBO operates by well-known concepts in chemistry such as hybridization, conjugation, hyper-conjugation, charge transfer, and orbital interactions. Natural energy decomposition analysis (NEDA) can be used for analysing intermolecular interactions between host–guest species [43]. The latest implementation of NEDA takes the form of a five-term energy decomposition:

Δ*E* = Δ*E_ES_* + Δ*E_POL_* + Δ*E_CT_* + Δ*E_XC_* + Δ*E_DEF_* (4)

where the first term Δ*E_ES_* is an electrostatic (ES) contribution. It describes the interaction of the unperturbed monomer charge densities and therefore the interaction of the permanent multipoles of the monomer units [34]. The next term Δ*E_POL_* is a polarization component (POL) and it describes the extra electrostatic interaction on polarizing the charge densities of the separated fragments in the field of the other fragments when in complex [42]. The third term, Δ*E_CT_*, is a “charge transfer” (CT) component and the forth term Δ*E_XC_* is a “steric exchange” (EX) component. It is considered to represent Pauli exchange-type repulsions between filled orbitals (or the quasiclassical “Lennard-Jones repulsion” between hard-shell sphere atoms). Natural steric analysis expresses steric exchange repulsion as the energy difference due to orbital orthogonalization, in accordance with a well-established physical picture of “steric repulsions” [41,42,43] For the total steric exchange energy, both the individual bond energy changes of natural localized molecular orbital compositions (NLMO), followed by the pairwise steric interactions between orbitals on different centres [41,42,43]. The remaining Δ*E_DEF_* term is the "deformation" energy (DEF), which represents the difference between the energies of the perturbed and relaxed monomer densities.

The optimized geometries of Fc (both E and S conformers) were the same as those in gas phase [2], which were obtained using the density functional theory (DFT) based B3LYP theory, in conjunction with the recently developed basis set for the transition metal Fe, that is, the m6-31G (d) basis set [67]. Two Staler type basis sets which are embedded in the ADF package [40], that is, TZ2P and TZ2P+ [68] are employed, together with three quantum mechanical models, that is, HF, B3LYP and a recent M06-2X level of theory. The natural energy decomposition analysis (NEDA) was performed with NBO 6.0 binary code [69], which links to GAMESS-US 2014 R1 program [44]. Energetics analysis with the NBO deletions was performed with the NBO 6.0 binary code linked to Gaussian 09 computational chemistry package [70]. All other calculations were based on the B3LYP/m6-31G(d) optimized structures [2] of E-Fc and S-Fc, and were performed using the Gaussian09.

## 3. Results and Discussion

### 3.1. ETS: EDA Dependence on the Fragmentation Channels of Fc

It is accepted that the eclipsed (D_5h_) ferrocene (E-Fc) exhibits a slightly lower total energy than the staggered ferrocene (D_5d_) [2,4,38,71,72,73] (at 0 K). However, in many cases the staggered, D_5d_ conformer of ferrocene has been employed for analyses [7,8,21,32], as it is often observed in room temperature comparison with other transition metal complexes [21]. Many previous studies of ferrocene were largely based on the qualitative MO diagram of staggered (D_5d_) ferrocene [21,24], which the electron configurations are largely model dependent as indicated in our previous studies [2,3] and in agreement with many other accurate calculations [37].

Although in principle one cannot go wrong when choosing a particular decomposition scheme in EDA [45] the interaction energies of the same complex can significantly depend on the fragmentation channels [20,21,22,23,24,36,38,46,74] within the same EDA scheme. Table 1 summarizes the previous EDA studies of ferrocene using different models in the ETS scheme. A number of fragmentation channels of ferrocene [21,24,38] are available: the ionic (heterolytic) channels consist of (i) ferrous Fe^2+^ cation with the valence electron configuration (a_1g_)^2^(e_2g_)^4^(e_1g_)° and (Cp^−^)_2_ anion [21]; (ii) the singly charged FeCp^+^ cation and singly charged Cp^−^ anion [20]; and (iii) the neutral (homolytic) channel formed by the neutral Fe and the neutral Cp_2_ as interacting fragments. In the homolytic channel, Fe is in a valence electron configuration triplet (a_1g_)^2^(e_2g_)^4^(e_1g_)^αα^, and the ligand Cp_2_ has the valence occupation (a_1g_)^2^(a_2u_)^2^(e_1u_)^4^(e_1g_)^ββ^ [21] The three energy components, that is, the Δ*E_Pauli_*, Δ*E_Elstat_* and Δ*E_Orb_*_,_ of the same ferrocene conformer are different, although the other conditions are the same, e.g., BP86/TZP.

**Table 1:** Summary of previous EDA studies for ferrocene conformers in the literature (kcal·mol^−1^) *.

Frag Channel	M + 2Cp	M^2+^ + 2Cp^−^	M^2+^ + 2Cp^−^	M^2+^ + 2Cp^−^	M^2+^ + 2Cp^−^	M^2+^ + 2Cp^−^	M^2+^ + Cp_2_^2−^	M^2+^ + Cp_2_^2−^	MCp^+^ + Cp^−^
Model Basis Set	BP86 TZP [21]	BP86 TZP [21]	BP86 TZP [22]	BP86 TZ2P [36]	B_PW91 TZ2P ^a^ [39]	OPBE TZP [37]	PW91 TZ2P [38]	PW91 TZ2P [38]	BP86 TZP [20]
Conformer	D_5d_	D_5d_	D_5d_	D_5d_	D_5h_	D_5h_	D_5h_	D_5d_	D_5d_
Δ*E_Pauli_*	409.6	279.9	272.2	282.0	–	345.7	399.23	398.85	172.4
Δ*E_elstat_*	−307.5	−599.9	−598.0	−600.5	–	−619.1	−229.05	−229.36	−238.5
Δ*E_orb_*	−376.3	−573.9	−567.5	−577.6	–	−634.5	−626.35	−624.17	−171.5
Δ*E_int_*	−274.2	−893.9	−893.3	−896.0	−919	−907.9	−456.17	−454.68	−237.6

* Based on the ADF computational chemistry package; ^a^ A modified Slater type TZ2P basis set (see [39]).

The fragmentation channel dependant differences of the energy components of ferrocene are nowhere close to each other. For example, the Δ*E_Pauli_* term (staggered Fc) is calculated using the BP86/TZP model as 272.2 kcal·mol^−1^ by Lein *et al.* [22], 172.4 kcal·mol^−1^ by Frunzke *et al.* [20], and 409.6 kcal·mol^−1^ by Rayón and Frenking [21] for the fragmentation channels (i), (ii) and (iii), respectively. In the same manner, the Δ*E_Elstat_* term is calculated as −598.9 kcal·mol^−1^ by Lein *et al.* [22] −238.5 kcal·mol^−1^ by Frunzke *et al.* [20] and −307.5 kcal·mol^−1^ by Rayón and Frenking [21] for the same Fc complex; whereas the Δ*E_Orb_* term in Equation (2) is calculated as −567.5 kcal·mol^−1^ by Lein *et al.* [22], −171.5 kcal·mol^−1^ by Frunzke *et al.* [20] and −376.3 kcal·mol^−1^ by Rayón and Frenking [21], respectively. Perhaps, what is in common (if any) in the three fragmentation channels is that the Δ*E_Pauli_* term is positive, whereas the ΔE_Elstat_ and ΔE_Orb_ terms are both negative. As indicated by Swart [37], the fragmentation channels will influence the interaction (bonding) energy significantly and the energy components. The fragmentation channels differ from the central metal Fe electrons, indicating the Fe-electrons play a significant role in the interaction energy and therefore the properties of the complex.

### 3.2. ETS: EDA Dependence on Quantum Mechanical Models Employed

In addition to fragmentation channels, the quantum mechanical models employed also impose large component energy differences, which make the EDA quite difficult for comparison and explanation purposes. For example, Swart [37] using the same fragmentation channel, *i.e.*, ferrous Fe^2+^ cation and (Cp^−^)_2_ anion (i) and the same basis set of triple zeta polarized (TZP) in ADF, but OPBE (combination of Handy's optimized exchange (OPTX) with the PBE correlation) rather than BP86 for the eclipsed ferrocene, the calculated energy components are apparently different [39]. As a result, such diverse results make it difficult to reveal whether the energy component differences between the eclipsed and staggered ferrocene are due to the model or due to the conformers.

In order to obtain a quantitative and consistent analysis for the interaction energies of ferrocene eclipsed (D_5h_) and staggered (D_5d_) conformers, one needs a consistent study based on the same conditions including the fragmentation channels and models. From the results summarized in Table 1, it is clear that the EDA energy components of the same conformer significantly depends on the fragmentation channels as well as the computational chemistry model, while other conditions are the same. To reduce the impact of the fragmentation schemes, the present study employed a “completed atomic scheme” FeCp_2_ → Fe ((3d)^6^, singlet) + 10 C ((2p)^2^, singlet) + 10 H ((1s)^1^, doublet), that is, the complex fragments into the smallest possible neutral atoms. Note that the atomic states are not necessarily the ground states of the atoms.

Table 2 further compares the EDA component energies calculated using the same DFT method of BP86 with different basis sets. It is seen from this table that, once the fragment channel is the same, there are very small or no differences between the Slater type basis sets, TZ2P, TZP and TZ2P+ [68], if the same DFT based (BP86) method is used. To calibrate the calculated energies, the HF/TZ2P model is also employed to calculate the same energy components at the HF level and ΔΔ*E* column (ΔΔ*E* = Δ*E*(D_5h_) − Δ*E*(D_5d_)) is the energy differences between the eclipsed and staggered conformers of ferrocene. The results from Zhang *et al.* [38] are also included as references as both conformers were available, but note these results are based on a different fragmentation channel (Fe^2+^ + Cp_2_^2−^) using the PW91/TZ2P model.

Table 2 reveals that the basis set effects to the energy components between the ferrocene conformers are small. The energy component changes ΔΔ*E* between the eclipsed and staggered ferrocene conformers do not vary apparently with respect to the basis sets. The calculated electrostatic energies, Δ*E_Estat_* for D_5h_, are basically the same using the same theory (BP86) among the basis sets, *i.e.*, both are −2794.94 kcal·mol^−1^, for TZP and TZ2P, respectively, but a slightly less negative energy of −2793.10 kcal·mol^−1^ is found when the TZ2P+ basis set is used for the same conformer. Similar trends are found for the Δ*E_Pauli_* but repulsive, *i.e.*, 12679.83 kcal·mol^−1^ when both TZP and TZ2P basis sets are used. As pointed by Bickelhaupt and Baerends [75], the basis set superposition errors (BBSE) are very small for the large Slater type basis sets used in the present study. Therefore, the energy contribution as a result of the formation of the electron-pair bond is not contained in ΔE_Pauli_ and ΔE_Elstat_ [75].

**Table 2:** Comparison of energy terms for the eclipsed (D_5h_) and staggered (D_5d_) ferrocene using the DFT based BP86 model and HF model with different basic sets (kcal·mol^−1^) ^1,2^.

Energy Frag ^1,2^	BP86/TZP			BP86/TZ2P			BP86/TZ2P+		
	D_5h_	D_5d_	ΔΔ*E_i_* ^3^	D_5h_	D_5d_	ΔΔ*E_i_* ^3^	D_5h_	D_5d_	ΔΔ*E_i_* ^3^
Δ*E_Estat_*	−2794.94	−2789.68	−5.26	−2794.94	−2789.68	−5.26	−2793.10	−2787.85	−5.25
Δ*E_Pauli_*	12,679.83	12,666.40	13.43	12,679.83	12,666.40	13.43	12,675.76	12,662.35	13.41
Δ*E_Orb_*	−12,942.17	−12,933.04	−9.13	−12,954.25	−12,945.16	−9.09	−12,952.85	−12,943.81	−9.04
Δ*E_Int_*	−3057.29	−3056.32	−0.97	−3069.36	−3068.45	−0.91	−3070.20	−3069.31	−0.89
Δ*E_Ster_*	9884.88	9876.71	8.17	9884.89	9876.71	8.18	9882.66	9874.50	8.16
**Energy Frag ^1,2^**	**HF/TZ2P ^4^**			**PW91/TZ2P ^5^**		
	**D_5h_**	**D_5d_**	**ΔΔ*E_i_*^3^**	**D_5h_**	**D_5d_**	**ΔΔ*E_i_*^3^**
Δ*E_Estat_*	−3332.24	−3325.18	−7.06	−229.05	−229.36	0.31
Δ*E_Pauli_*	14,486.28	14,470.29	15.99	−399.23	−398.85	−0.38
Δ*E_Orb_*	−16,465.98	−16,457.79	−8.19	−626.35	−624.17	−2.18
Δ*E_Int_*	−5311.94	−5312.68	0.74	−456.17	−454.68	−1.49
Δ*E_Ster_*	11,154.04	11,145.11	8.93	−628.28	−628.21	−0.07

^1^ The fragment scheme is Fe(C_5_H_5_)_2_ → Fe ((3d)^6^, singlet) + 10 C ((2p)^2^, singlet) + 10 H ((1s)^1^, doublet); ^2^ Based on the B3LYP/m6-31G(d) optimised geometry [2]; ^3^ ΔΔ*E_i_* = Δ*E_i_*(D_5h_) − Δ*E_i_*(D_5d_); ^4^ In the HF/TZ2P EDA calculations, the QZ4P fit was applied [40]; ^5^ The fragmentation scheme is Fe(C_5_H_5_)_2_ → M^2+^ + Cp_2_^2−^ [38].

The small basis set effects on energy components show in the orbital energy term, ΔΔ*E_Orb_*_,_ as this energy term is responsible for orbital interaction or relaxation [75]. This term is the basis set dependent charge-transfer and polarization (and mix) term [75]. The energy differences, ΔΔ*E_i_*, for the electrostatic energy and the orbital energy between the eclipsed and staggered ferrocene conformers exhibit negative values, which are balanced but the almost twice as large Pauli positive energy values, leading to the total interaction energy change, ΔΔ*E_Int_* a very small energy value. For example, using the BP86/TZ2P+ model, the ΔΔ*E* components for ΔΔ*E_Pauli_*, ΔΔ*E_Elstat_* and ΔΔ*E_Orb_*, are given by 13.41 kcal·mol^−1^, −5.25 kcal·mol^−1^ and −9.04 kcal·mol^−1^, respectively, which results in the interaction energy ΔΔ*E_Int_* (See Equation (2)) of only −0.89 kcal·mol^−1^. The results in Table 2 show that under the same theory, *i.e.*, BP86, the larger the basis set, the slightly less negative the total interaction energy difference between the eclipsed (E-Fc) and staggered (S-Fc) ferrocene conformers. For example, the absolute value of ΔΔE_Int_ changes from −0.97 kcal·mol^−1^ (BP86/TZP), to −0.91 kcal·mol^−1^ (BP86/TZ2P) and to −0.89 kcal·mol^−1^ (BP86/TZ2P+). Hence, under the same fragmentation scheme (in the present study, the fragment scheme is completely dissociating into neutral atoms), the energy difference of the electrostatic energy between E-Fc and S-Fc remains at −5.25 kcal·mol^−1^ across the basis sets, followed by the energy term of orbital interaction changes of ΔΔ*E_Orb_* with −9.04 kcal·mol^−1^ when TZ2P+ basis set is used. The largest energy change between the conformers is the repulsive Pauli (or steric) energy component, which is as large as 13.41 kcal·mol^−1^ using the same model of BP86/TZ2P+.

It is also interesting to compare the ΔΔEs among BP86/TZ2P, HF/TZ2P and PW91/TZ2P models. As the BP86/TZ2P and HF/TZ2P models are based on the same fragment channel, the energy differences can be attributed to inclusion of electron correlation of the BP86 method. The missing electron correlation energy contributes to increase the ΔΔ*E_Elstat_* (more negative), ΔΔ*E_Pauli_*, and ΔΔ*E_Ster_*, but decrease the ΔΔ*E_Orb_*. As a result, the total interaction energy, ΔΔ*E_Int_* changes its sign from preferring the eclipsed Fc (in BP86/TZ2P) to preferring the staggered Fc (HF/TZ2P). In addition, the heterolytic fragmentation channel of Zhang *et al.* [38] (Fe^2+^ + Cp_2_^2−^) in the PW91/TZ2P model indicates that the pre-partitioned fragments of the ferrocene conformers, although largely changed the preference of individual energy component, the total interaction energy ΔΔ*E_Int_*, again, prefers the eclipsed conformer. It also indicated that the energies have already been absorbed into the pre-partitioned fragments of the conformers.

The decomposed energy terms of a molecule also depends on the theory employed [74]. Table 3 compares the EDA energies using different level of theory combining with the TZ2P+ basis set. This TZ2P+ is a Slater basis set that is close to the modified Gaussian basis set of m6-31G(d) [67]. Four levels of theory are employed, that is, HF, BP86, M06-2X and B3LYP. Similar trend to the results of basis sets in Table 2 is observed. The electrostatic energy and the orbital energy between the E-Fc and S-Fc conformers exhibit attractive negative values, which are nearly balanced but the almost twice as large positive Pauli energy values, leading to the total interaction energy change, ΔΔ*E_Int_* a very small energy value. The energy differences obtained from DFT models in the same table are very small with respect to the HF/TZ2P+ model. For example, the total interaction energy differences (ΔΔ*E_Int_*) are given by 1.60 kcal·mol^−1^, 0.76 kcal·mol^−1^, −0.09 kcal·mol^−1^ and −0.89 kcal·mol^−1^ from the HF, M06-2X, B3LYP and BP86, respectively. The HF/TZ2P+ model gives the ΔΔ*E_Pauli_*, ΔΔ*E_Elstat_* and ΔΔ*E_Orb_* terms as 14.62 kcal·mol^−1^, −5.25 kcal·mol^−1^ and −7.76 kcal·mol^−1^, respectively. The HF results under the same conditions reveal that the largest relative energy changes with respect to the BP86 model between the E-Fc and S-Fc is the ΔΔ*E_Orb_* term with 16.5%, and followed by the ΔΔ*E_Pauli_* term with −8.3% and the ΔΔ*E_Elstat_* term remain unchanged. The total steric energy between the E-Fc and S-Fc conformers becomes smaller when electron correlation effect is taken into account, as this energy term ΔΔ*E_Steric_* is reduced from 9.36 kcal·mol^−1^ in the HF/TZ2P+ model to 8.40 kcal·mol^−1^ in the B3LYP/TZ2P+ model.

**Table 3:** Comparison of energy terms for the eclipsed (D_5h_) and staggered (D_5d_) ferrocene using different level of theory with the TZ2P+ basic set (kcal·mol^−1^) ^1,2^.

**Energy Terms ^1,2^**	**HF/TZ2P+**			**M06-2X/TZ2P+**		
	**D_5h_**	**D_5d_**	**ΔΔ*E_i_*^3^**	**D_5h_**	**D_5d_**	**ΔΔ*E_i_*^3^**
Δ*E_Estat_*	−2793.10	−2787.85	−5.25	−2793.10	−2787.85	−5.25
Δ*E_Pauli_*	−49,351.36	−49,365.98	14.62	−54,025.19	−54,039.14	13.95
Δ*E_Orb_*	−16,840.51	−16,832.75	−7.76	−14,568.75	−14,560.82	−7.93
Δ*E_Int_*	−68,984.98	−68,986.58	1.60	−71,387.05	−71,387.81	0.76
Δ*E_Ster_*	−52,144.47	−52,153.83	9.36	−56,818.3	−56,826.99	8.69
**Energy Terms ^1,2^**	**B3LYP/TZ2P+**			**BP86/TZ2P+**		
	**D_5h_**	**D_5d_**	**ΔΔ*E_i_*^3^**	**D_5h_**	**D_5d_**	**ΔΔ*E_i_*^3^**
Δ*E_Estat_*	−2793.10	−2787.85	−5.25	−2793.10	−2787.85	−5.25
Δ*E_Pauli_*	222.39	208.73	13.66	12,675.76	12,662.35	13.41
Δ*E_Orb_*	−13,630.90	−13,622.42	−8.48	−12,952.85	−12,943.81	−9.04
Δ*E_Int_*	−16,201.62	−16,201.53	−0.09	−3070.20	−3069.31	−0.89
Δ*E_Ster_*	−2570.72	−2579.12	8.40	9882.66	9874.50	8.16

^1^ The fragment scheme is Fe(C_5_H_5_)_2_ → Fe ((3d)^6^, singlet) + 10 C ((2p)^2^, singlet) + 10 H ((1s)^1^, doublet); ^2^ based on the B3LYP/m6-31G(d) optimised geometry [2]; ^3^ ΔΔE_i_ = ΔE_i_(D_5h_) − ΔE_i_(D_5d_).

The B3LYP/m6-31G(d) is superior in not only producing an accurate result in IR spectrum [2] and geometry [3] of ferrocene, but also in providing a consistently accurate description of the heterolytic dissociation enthalpy of the complex [76], the discussion of the present study will concentrate on the results produced by the B3LYP/TZ2P+ model from the atomic fragmentation scheme. The energy differences between the terms, ΔΔ*E_Pauli_*, ΔΔ*E_Elstat_* and ΔΔ*E_Orb_*, are 13.66 kcal·mol^−1^, −5.25 kcal·mol^−1^, and −8.48 kcal·mol^−1^, respectively. As pointed out by Bickelhaupt and Baerends [75] in their earlier study of conformation of ethane, the Pauli repulsive energy is always higher when going to the eclipsed conformation with all other geometry parameters fixed, in agreement with the ferrocene eclipsed conformer with ΔΔ*E_Pauli_* =13.66 kcal·mol^−1^ higher energy than the staggered conformer. The electrostatic interaction is more attractive in the eclipsed conformer (see Table 3) as ΔΔ*E_Elstat_* = −5.25 kcal·mol^−1^. The ΔΔ*E_Elstat_* term indicates that the charge distribution of the atomic fragments leads not only to higher steric interaction, but also more attractive electrostatic interaction [75]. In general, the total electrostatic energy is attractive (negative) since the electron-nucleus attraction outweighs the repulsive terms. Obviously, the eclipsed ferrocene is slightly more attractive than the staggered complex. As a result, the origin of steric repulsion is not electrostatic repulsion between electrons and nuclei, but favours steric three-dimensional positions, whereas the steric repulsion is originated from the quantum mechanical Pauli exclusion energy [75]. The orbital interaction energy change of Fc, ΔΔ*E_Orb_* = −8.48 kcal·mol^−1^, is more attractive when the overlap of the charge clouds is larger, such as in the eclipsed Fc. This is because that the orbitals overlaps between occupied-occupied and occupied-unoccupied orbitals are all larger which leads to stronger donor–acceptor interactions [75].

Conformational isomers such as n-butane [77] and ethane [75] usually take the staggered conformer as the global minimum structure, due to the steric hindrance [75]. However, in the case of ferrocene the Cp rings are separated through bonding with a transition metal Fe. From Table 3 it is seen that the total interaction energy difference between the conformers of ferrocene, ΔΔ*E_Int_*, is a small residue of the balance between Pauli repulsive term ΔΔ*E_Pauli_* and the attractive electrostatic and orbital energies of ΔΔ*E_Elstat_* + ΔΔ*E_Orb_*. Interestingly, the electrostatic energy term in this table ΔΔ*E_Elstat_* is independent of the level of theory, as the HF and the DFT models produce the same electrostatic energy. As a result, the major difference between the Fc conformers is due to the quantum mechanical Pauli repulsive energy and orbital attractive energy, in agreement with chemical intuition.

### 3.3. NBO-NEDA Analysis of Ferrocene Conformers

To better understand the nature of interactions in ferrocene conformers in terms of meaningful physical components we applied the natural energy decomposition analysis (NEDA) scheme using the B3LYP/m6-31G(d) optimized geometries [2], which have been employed in the previous ETS section. Table 4 compares contributions of the decomposed energy terms based on the NEDA scheme for the eclipsed and staggered conformers of ferrocene. Comparing the data shows that the main driving forces responsible for the higher relative stability of eclipsed conformer are mainly electrical and charge transfer (CT) interactions, which account for −4.82 and −5.35 kcal·mol^−1^, respectively. We may notice a relatively large contribution of the polarization interaction (−6.27 kcal·mol^−1^) into the higher relative stability of the eclipsed conformer. These strong electric and charge transfer (CT) interactions are necessary to overcome the strong CORE repulsion, which is 9.73 kcal·mol^−1^ higher than the staggered conformation.

**Table 4:** Energy decomposition of ferrocene conformational differences using NEDA scheme (kcal·mol^−1^) ^a,b^.

Energy Component ^c^	D_5h_	D_5d_	ΔE ^d^
EL (ES + POL + SE)	−1011.05	−1006.23	−4.82
ES	−676.89	−675.31	−1.58
POL	−671.73	−665.46	−6.27
SE (Fe)	35.89	35.84	0.05
SE (Cp)	150.84	149.35	1.49
SE (Cp)	150.84	149.35	1.49
Tot SE	337.57	334.54	3.03
Charge Transfer (CT)	−634.02	−628.67	−5.35
Core (XC + DEF − SE)	880.64	870.91	9.73
XC	−200.44	−198.58	−1.86
DEF (Fe)	395.28	392.47	2.81
DEF (Cp)	511.69	505.78	5.91
DEF (Cp)	511.68	505.78	5.90
Tot DEF	1418.65	1404.03	14.62
Etot (EL + CT + Core)	−764.43	−763.99	−0.44

^a^ using B3LYP/m6-31G(d) model; ^b^ The fragment scheme is FeCp_2_ → Fe + 2Cp; ^c^ ES: electrostatic; POL: polarization; SE: self-energy correction (“polarization penalty”) for each centre; XC: exchange; DEF: “deformation” energy; ^d^ Δ*E* = *E*(D_5h_) − *E*(D_5d_);

The NBO 6.0 program [69] makes it possible to investigate the effect of specific natural bond orbital (NBO) donor–acceptor interactions on the conformer’s energy difference. It uses the so-called “deletions” when certain NBOs, group of NBOs, or specific NBO donor–acceptor interactions can be deleted to determine their energetic effects. For example, to estimate the role of electronic delocalization on the conformer’s stability we can delete all non-Lewis NBOs from the basis set, with the resulting “natural Lewis structure” wave function being perfectly localized and with all Lewis-type NBOs doubly occupied. In this case the stabilizing effect of the delocalizing (non-Lewis) contributions, *E_NL_*, will be expressed as

*E_NL_* = *E*(full) − *E_L_* (5)

where *E*(full) is the original energy and *E_L_* is the Lewis-type localized energy.

The NBO analysis further indicates that while the Lewis (*E_L_*) energy which prefers the eclipsed Fc and the non-Lewis (*E_NL_*) energy which favours staggered Fc, the total energy between the eclipsed and staggered Fc largely cancels out with a relatively small residue. As one can see in Table 5 (section *Lewis Contribution*) the localized E_L_ contribution strongly favours the eclipsed (D_5h_) configuration by 564.4 kcal·mol^−1^, while the delocalization contribution *E_NL_* favours the staggered (D_5d_) conformer by a similar amount of 563.9 kcal·mol^−1^, leading to nearly equal total energies for the two conformers with a small residue of −0.52 kcal·mol^−1^. Thus, the stability of the staggered conformer can therefore be attributed to the electronic delocalization energy *E_NL_*. In the absence of the Fe atom (section *Lewis Contribution (NoFe)*) in Table 5, we see a similar picture but *E_L_* and *E_NL_* contributions are much smaller (−23.3 and 23.1 kcal·mol^−1^, correspondingly), with the difference between two conformations being also smaller (−0.18 kcal·mol^−1^). However, the Lewis contributions with and without Fe in Table 5 indicate that the significant localised (Lewis) and delocalised (non-Lewis) contributions of Fc conformers are due to the orientations of the cyclopentadienyle (Cp) rings, as only approximately ±23 kcal·mol^−1^ of such energies are due to the Fe atom, which counts for less 5% of the (non-)Lewis contributions.

**Table 5:** NBO Analysis of ferrocene conformational differences *.

Energy Decomposition	*E*(D_5h_)/*E_h_*	*E*(D_5d_)/*E_h_*	Δ*E* ^a^/ kcal·mol^−1^
Lewis Contribution (Fc)			
Total	−1650.66185	−1650.661026	−0.52
Total Lewis (*E_L_*)	−1648.30198	−1647.402542	−564.41
Total Non-Lewis (*E_NL_*)	−2.35987395	−3.25848317	563.89
Lewis Contribution (No Fe)			
Total	−386.897706	−386.8974192	−0.18
Total Lewis (*E_L_*)	−385.653328	−385.6162447	−23.27
Total Non-Lewis (*E_NL_*)	1.244378	1.281174	23.09
LP(Fe) – BD *(C-C & C-H)			
Energy of Deletion (*E_D_*)	−1650.02738	−1649.298117	−457.62
LP(Fe) – RY *(C & H)			
Energy of Deletion (*E_D_*)	−1650. 02384	−1650.013585	−6.43

* Based on B3LYP/m6-31G(d) optimized geometries [3]; ^a^ Δ*E* = *E*(Eclipsed) − *E* (Staggered).

We can further dissect the delocalizing (non-Lewis) contribution, *E_NL_*, into specific donor–acceptor contributions by, for example, exploratory deletion of all interactions between the donor lone pairs (LP) of the iron atom and the acceptor antibond (BD*) NBOs of all C–C and C–H bonds, which is given in section LP(Fe) − BD*(C-C & *C-H)* of Table 5. As we can see a deletion of these donor–acceptor interactions strongly destabilizes the staggered (D_5d_) conformation by 457.6 kcal·mol^−1^. It indicates that a significant energy difference between the eclipsed and staggered ferrocene is caused by the interactions between the lone pair of Fe and the antibond electrons of the orientation of the Cp rings, *i.e.*, the conformers. On the other hand, the donor–acceptor interactions involving the donor lone pairs (LP) of Fe atom and the unoccupied Rydberg-type (RY*) acceptor NBOs of the carbon and hydrogen atoms lead to a relatively small destabilization of the staggered (D_5d_) conformer by 6.4 kcal·mol^−1^, as given in section LP(Fe) − RY*(C & *H)* of Table 5.

## 4. Conclusions

Quantum mechanically based energy decomposition analyses (EDA) are employed to study interaction energies of ferrocene conformers. Two very different EDA schemes are employed with one based on the extended transition state (ETS) using the ADF package and the other on natural EDA (NEDA) using the GAMESS package. The study reveals that the interaction energy terms of ferrocene depend on a number of factors, not only the model (theory and basis set) but also more significantly, the fragmentation schemes in the EDA. It is discovered that interaction energy can be absorbed into the pre-partitioned fragments such as the heterolytic fragmentation, which may affect the interaction energies in a particular conformer of Fc. The present study employs a complete individual atomic fragment scheme—the fragments of ferrocene are neutral atoms rather than pre-partitioned fragments. In the ETS scheme, it discovers that unlike the alkane rotational conformers such as n-butane and ethane, which the steric hindrance results in the staggered conformer as the energy favoured structure, the major difference between the ferrocene conformers is due to the quantum mechanical Pauli repulsive energy and orbital attractive energy, leading to the eclipsed ferrocene the energy preferred structure.

The NEDA scheme further indicates the attractive (negative) polarization (POL) and charge transfer (CL) energies prefer the eclipsed ferrocene. The repulsive (positive) deformation (DEF) energy, which is dominated by the cyclopentadienyle (Cp) rings, prefers the staggered ferrocene. The polarization (POL) and charge transfer (CL) energies are nearly balanced out by the deformation (DEF) energy, leading to small attractive total interaction energy residue preferring the eclipsed Fc conformer. Further NBO analysis indicates that the localized Lewis *E_L_* contribution strongly favours the eclipsed (D_5h_) configuration by 564.4 kcal·mol^−1^, while the delocalization non-Lewis contribution *E_NL_* favours the staggered (D_5d_) conformer by a similar amount of 563.9 kcal·mol^−1^ leading to nearly equal total energies for the two conformers with a small residue of −0.52 kcal·mol^−1^, in favour of the eclipsed conformer. The most significant energy between the eclipsed and staggered ferrocene which is not cancelled out is the deletion of all interactions between the donor lone pairs (LP) of the iron atom and the acceptor antibond (BD*) NBOs of all C-C and C-H bonds (LP(Fe) − BD*(C-C & *C-H)*), which strongly destabilizes the staggered (D_5d_) conformation by 457.6 kcal·mol^−1^.

## References

[1] Kealy T.J., Pauson P.L. (1951). A new type of organo-Iron compound. Nature.

[2] Mohammadi N., Ganesan A., Chantler C.T., Wang F. (2012). Differentiation of ferrocene D5d and D5h conformers using IR spectroscopy. J. Organomet. Chem..

[3] Islam S., Wang F. (2015). The d-electrons of Fe in ferrocene: The excess orbital energy spectrum (EOES). RSC Adv..

[4] Haaland A., Nilsson J.E. (1968). The determination of barriers to internal rotation by means of electron diffraction. Ferrocene and ruthenocene. Acta Chem. Scand..

[5] Haaland A., Lusztyk J., Novak D.P., Brunvoll J., Starowieyski K.B. (1974). Molecular structures of dicyclopentadienylmagnesium and dicyclopentadienylchromium by gas-phase electron diffraction. J. Chem. Soc. Chem. Commun..

[6] Haaland A. (1979). Molecular structure and bonding in the 3d metallocenes. Acc. Chem. Res..

[7] Lippincott E.R., Nelson R.D. (1953). The vibrational spectra and structure of ferrocene and ruthenocene. J. Chem. Phys..

[8] Lippincott E.R., Nelson R.D. (1958). The vibrational spectra and structure of ferrocene and ruthenocene. Spectrochim. Acta.

[9] Cotton F.A., Wilkinson G., Murillo C.A., Bochmann M. (1988). Advanced Inorganic Chemistry.

[10] Yamaguchi Y., Ding W., Sanderson C.T., Borden M.L., Morgan M.J., Kutal C. (2007). Electronic structure, spectroscopy, and photochemistry of group 8 metallocenes. Coord. Chem. Rev..

[11] Duhović S., Diaconescu P.L. (2013). An experimental and computational study of 1,1′-ferrocene diamines. Polyhedron.

[12] Cooper D.C., Yennie C.J., Morin J.B., Delaney S., Suggs J.W. (2011). Development of a DNA-damaging ferrocene amino acid. J. Organomet. Chem..

[13] Gryaznova T.P., Katsyuba S.A., Milyukov V.A., Sinyashin O.G. (2010). DFT study of substitution effect on the geometry, IR spectra, spin state and energetic stability of the ferrocenes and their pentaphospholyl analogues. J. Organomet. Chem..

[14] Mehrotra R.C., Singh A. (2007). Organometallic Chemistry.

[15] Reddy K.V. (1998). Symmetry and Spectroscopy of Molecules.

[16] Atkins P., Overton T., Rourke J., Weller M., Armstrong F. (2010). Shriver and Atkins' Inorganic Chemistry.

[17] Coriani S., Haaland A., Helgaker T., Jørgensen P. (2006). The Equilibrium Structure of Ferrocene. ChemPhysChem.

[18] Roy D.R., Duley S., Chattaraj P.K. (2008). Bonding, reactivity and aromaticity in some novel all-metal metallocenes. Proc. Indian Natl. Sci. Acad. Phys. Sci..

[19] Bean D.E., Fowler P.W., Morris M.J. (2011). Aromaticity and ring currents in ferrocene and two isomeric sandwich complexes. J. Organomet. Chem..

[20] Frunzke J., Lein M., Frenking G. (2002). Structures, metal-ligand bond strength, and bonding analysis of ferrocene derivatives with group-15 heteroligands Fe(η5-E5)2 and FeCp(η5-E5) (E = N, P, As, Sb). A Theoretical Study. Organometallics.

[21] Rayón V.M., Frenking G. (2003). Bis(benzene)chromium is a δ-bonded molecule and ferrocene is a π-bonded molecule. Organometallics.

[22] Lein M., Frunzke J., Timoshkin A., Frenking G. (2001). Iron bispentazole Fe(η5-N5)2, a theoretically predicted high-energy compound: Structure, bonding analysis, metal–ligand bond strength and a comparison with the isoelectronic ferrocene. Chem. Eur. J..

[23] Frenking G., Fröhlich N. (2000). The nature of the bonding in transition-metal compounds. Chem. Rev..

[24] Frenking G., Wichmann K., Fröhlich N., Loschen C., Lein M., Frunzke J., Rayón V.C.M. (2003). Towards a rigorously defined quantum chemical analysis of the chemical bond in donor-acceptor complexes. Coord. Chem. Rev..

[25] Cortés-Guzmán F., Bader R.F.W. (2005). Complementarity of QTAIM and MO theory in the study of bonding in donor-acceptor complexes. Coord. Chem. Rev..

[26] Xu Z.-F., Xie Y., Feng W.-L., Schaefer H.F. (2003). Systematic investigation of electronic and molecular structures for the first transition metal series metallocenes M(C5H5)2 (M = V, Cr, Mn, Fe, Co, and Ni). J. Phys. Chem. A.

[27] Salzner U. (2010). Modeling photoelectron spectra of conjugated oligomers with time-dependent density functional theory. J. Phys. Chem. A.

[28] Zlatar M., Schläpfer C.-W., Daul C., Köppel H., Yarkony D.R., Barentzen H. (2009). A New Method to Describe the Multimode Jahn-Teller Effect Using Density Functional Theory. The Jahn-Teller Effect.

[29] Wilkinson G., Rosenblum M., Whiting M.C., Woodward R.B. (1952). The structure of iron bis-cyclopentadienyl. J. Am. Chem. Soc..

[30] Bohn R.K., Haaland A. (1966). On the molecular structure of ferrocene, Fe(C_5_H_5_)_2_. J. Organomet. Chem..

[31] Fischer V.E.O., Pfab W. (1952). Cyclopentadien-metallkomplexe, ein neuer Typ metallorganischer verbindungen. Z. Naturforschung.

[32] Lippincott E.R., Nelson R.D. (1955). The thermodynamic functions of bis-cyclopentadienyl iron, bis-cyclopentadienylnickel and bis-cyclopentadienylruthenium. J. Am. Chem. Soc..

[33] Ziegler T., Rauk A. (1977). On the calculation of bonding energies by the Hartree Fock Slater method. Theoret. Chim. Acta.

[34] Schenter G.K., Glendening E.D. (1996). Natural energy decomposition analysis: The linear response electrical self energy. J. Phys. Chem..

[35] Morokuma K. (1971). Molecular Orbital Studies of hydrogen bonds. III. C=O···H–O hydrogen bond in H_2_CO···H_2_O and H_2_CO···2H_2_O. J. Chem. Phys..

[36] Gomez-Sandoval Z., Peña E., Guerra C.F., Bickelhaupt F.M., Mendez-Rojas M.A., Merino G. (2009). A helicoid ferrocene. Inorg. Chem..

[37] Swart M. (2007). Metal-ligand bonding in metallocenes: Differentiation between spin state, electrostatic and covalent bonding. Inorg. Chim. Acta.

[38] Zhang G., Zhang H., Sun M., Liu Y., Pang X., Yu X., Liu B., Li Z. (2007). Substitution effect on the geometry and electronic structure of the ferrocene. J. Comput. Chem..

[39] Mayor-López M.J., Weber J. (1997). DFT calculations of the binding energy of metallocenes. Chem. Phys. Lett..

[40] Gusarov S., van den Hoek F.E.H.P., Jacob C.R., Jacobsen H., Jensen L., Kaminski J.W., van Kessel G., Kootstra F., Kovalenko A., Krykunov M.V. (2014). ADF2014.

[41] Glendening E.D., Streitwieser A. (1994). Natural energy decomposition analysis: An energy partitioning procedure for molecular interactions with application to weak hydrogen bonding, strong ionic, and moderate donor—Acceptor interactions. J. Chem. Phys..

[42] Glendening E.D. (1996). Natural energy decomposition analysis: Explicit evaluation of electrostatic and polarization effects with application to aqueous clusters of alkali metal cations and neutrals. J. Am. Chem. Soc..

[43] Glendening E.D. (2005). Natural energy decomposition analysis: Extension to density functional methods and analysis of cooperative effects in water clusters. J. Phys. Chem. A.

[44] Schmidt M.W., Baldridge K.K., Boatz J.A., Elbert S.T., Gordon M.S., Jensen J.H., Koseki S., Matsunaga N., Nguyen K.A., Su S. (1993). General atomic and molecular electronic structure system. J. Comput. Chem..

[45] Wolters L.P., Bickelhaupt F.M. (2015). The activation strain model and molecular orbital theory. Wiley Interdiscip. Rev. Comput. Mol. Sci..

[46] Hopffgarten M.V., Frenking G. (2012). Energy decomposition analysis. Wiley Interdiscip. Rev. Comput. Mol. Sci..

[47] Hirao H. (2007). Reactive bond orbitals: A localized resonance-structure approach to charge transfer. Chem. Phys. Lett..

[48] Ziegler T., Rauk A. (1979). A theoretical study of the ethylene-metal bond in complexes between copper(1+), silver(1+), gold(1+), platinum(0) or platinum(2+) and ethylene, based on the Hartree-Fock-Slater transition-state method. Inorg. Chem..

[49] Ziegler T., Rauk A. (1979). Carbon monoxide, carbon monosulfide, molecular nitrogen, phosphorus trifluoride, and methyl isocyanide as sigma. donors and pi. acceptors. A theoretical study by the Hartree-Fock-Slater transition-state method. Inorg. Chem..

[50] Su P., Li H. (2009). Energy decomposition analysis of covalent bonds and intermolecular interactions. J. Chem. Phys..

[51] Stevens W.J., Fink W.H. (1987). Frozen fragment reduced variational space analysis of hydrogen bonding interactions. Application to the water dimer. Chem. Phys. Lett..

[52] Mo Y., Gao J., Peyerimhoff S.D. (2000). Energy decomposition analysis of intermolecular interactions using a block-localized wave function approach. J. Chem. Phys..

[53] Mo Y., Bao P., Gao J. (2011). Energy decomposition analysis based on a block-localized wavefunction and multistate density functional theory. Phys. Chem. Chem. Phys..

[54] Misquitta A.J., Podeszwa R., Jeziorski B., Szalewicz K. (2005). Intermolecular potentials based on symmetry-adapted perturbation theory with dispersion energies from time-dependent density-functional calculations. J. Chem. Phys..

[55] Kitaura K., Morokuma K. (1976). A new energy decomposition scheme for molecular interactions within the Hartree-Fock approximation. Int. J. Quantum Chem..

[56] Khaliullin R.Z., Cobar E.A., Lochan R.C., Bell A.T., Head-Gordon M. (2007). Unravelling the origin of intermolecular interactions using absolutely localized molecular orbitals. J. Phys. Chem. A.

[57] Reed A.E., Curtiss L.A., Weinhold F. (1988). Intermolecular interactions from a natural bond orbital, donor-acceptor viewpoint. Chem. Rev..

[58] Jeziorski B., Moszynski R., Szalewicz K. (1994). Perturbation theory approach to intermolecular potential energy surfaces of van der waals complexes. Chem. Rev..

[59] Blanco M.A., Martín Pendás A., Francisco E. (2005). Interacting quantum atoms: A correlated energy decomposition scheme based on the quantum theory of atoms in molecules. J. Chem. Theory Comput..

[60] Mitoraj M.P., Michalak A., Ziegler T. (2009). A combined charge and energy decomposition scheme for bond analysis. J. Chem. Theory Comput..

[61] Szalewicz K. (2012). Symmetry-adapted perturbation theory of intermolecular forces. Wiley Interdiscip. Rev. Comput. Mol. Sci..

[62] Ziegler T., Tschinke V., Becke A. (1987). Theoretical study on the relative strengths of the metal-hydrogen and metal-methyl bonds in complexes of middle to late transition metals. J. Am. Chem. Soc..

[63] Ziegler T., Tschinke V., Ursenbach C. (1987). Thermal stability and kinetic lability of the metal carbonyl bond. A theoretical study on M(CO)6 (M = chromium, molybdenum, tungsten), M(CO)5 (M = iron, ruthenium, osmium), and M(CO)4 (M = nickel, palladium, platinum). J. Am. Chem. Soc..

[64] Li J., Schreckenbach G., Ziegler T. (1995). A reassessment of the first metal-carbonyl dissociation energy in M(CO)4 (M = Ni, Pd, Pt), M(CO)5 (M = Fe, Ru, Os), and M(CO)6 (M = Cr, Mo, W) by a quasirelativistic density functional method. J. Am. Chem. Soc..

[65] Ehlers A.W., Baerends E.J., Bickelhaupt F.M., Radius U. (1998). Alternatives to the CO ligand: Coordination of the isolobal analogues BF, BNH_2_, BN(CH_3_)_2_, and BO- in mono- and binuclear first-row transition metal complexes. Chem. Eur. J..

[66] Foster J.P., Weinhold F. (1980). Natural hybrid orbitals. J. Am. Chem. Soc..

[67] Mitin A.V., Baker J., Pulay P. (2003). An improved 6-31G* basis set for first-row transition metals. J. Chem. Phys..

[68] Chong D.P., Van Lenthe E., Van Gisbergen S., Baerends E.J. (2004). Even-tempered slater-type orbitals revisited: From hydrogen to krypton. J. Comput. Chem..

[69] Glendening E.D., Badenhoop J.K., Reed A.E., Carpenter J.E., Bohmann J.A., Morales C.M., Landis C.R., Weinhold F. (2013). NBO 6.0.

[70] Frisch M.J., Trucks G.W., Schlegel H.B., Scuseria G.E., Robb M.A., Cheeseman J.R., Scalmani G., Barone V., Mennucci B., Petersson G.A. (2009). Gaussian 09.

[71] Koch H., Jorgensen P., Helgaker T. (1996). The molecular structure of ferrocene. J. Chem. Phys..

[72] Hohm U., Goebel D., Grimme S. (1997). Experimental and theoretical study of the dipole polarizability of ferrocene Fe(C_5_H_5_)_2_. Chem. Phys. Lett..

[73] Pierloot K., Persson B.J., Roos B.O. (1995). Theoretical study of the chemical bonding in [Ni(C_2_H_4_)] and ferrocene. J. Phys. Chem..

[74] Vyboishchikov S.F., Krapp A., Frenking G. (2008). Two complementary molecular energy decomposition schemes: The Mayer and Ziegler–Rauk methods in comparison. J. Chem. Phys..

[75] Bickelhaupt F.M., Baerends E.J. (2003). The case for steric repulsion causing the staggered conformation of ethane. Angew. Chem. Int. Ed..

[76] Phung Q.M., Vancoillie S., Pierloot K. (2012). A Multiconfigurational perturbation theory and density functional theory study on the heterolytic dissociation enthalpy of first-row metallocenes. J. Chem. Theory Comput..

[77] Wang F. (2003). Assessment of quantum mechanical models based on resolved orbital momentum distributions of n-butane in the outer valence shell. J. Phys. Chem. A.

